# Editorial: plant-microbial symbiosis toward sustainable food security

**DOI:** 10.1080/15592324.2023.2298054

**Published:** 2024-01-05

**Authors:** Ixchel Campos-Avelar, Amelia C. Montoya-Martínez, Fannie I. Parra-Cota, Sergio de los Santos-Villalobos

**Affiliations:** a Laboratorio de Biotecnología del Recurso Microbiano, Instituto Tecnológico de Sonora (ITSON), Ciudad Obregon, Mexico; b Campo Experimental Norman E. Borlaug, Instituto Nacional de Investigaciones Forestales, Agrícolas y Pecuarias (INIFAP), Ciudad Obregon, Mexico

**Keywords:** Beneficial microorganisms, food security, microbial inoculants, soil fertility, sustainable agriculture

## Abstract

The use of plant-associated microorganisms is increasingly being investigated as a key tool for mitigating the impact of biotic and abiotic threats to crops and facilitating migration to sustainable agricultural practices. The microbiome is responsible for several functions in agroecosystems, such as the transformation of organic matter, nutrient cycling, and plant/pathogen growth regulation. As climate change and global warming are altering the dynamics of plant-microbial interactions in the ecosystem, it has become essential to perform comprehensive studies to decipher current and future microbial interactions, as their useful symbiotic mechanisms could be better exploited to achieve sustainable agriculture. This will allow for the development of effective microbial inoculants that facilitate nutrient supply for the plant at its minimal energy expense, thus increasing its resilience to biotic and abiotic stresses. This article collection aims to compile state-of-the-art research focused on the elucidation and optimization of symbiotic relationships between crops and their associated microbes. The information presented here will contribute to the development of next-generation microbial inoculants for achieving a more sustainable agriculture.

## GRAPHICAL ABSTRACT

**Figure:**
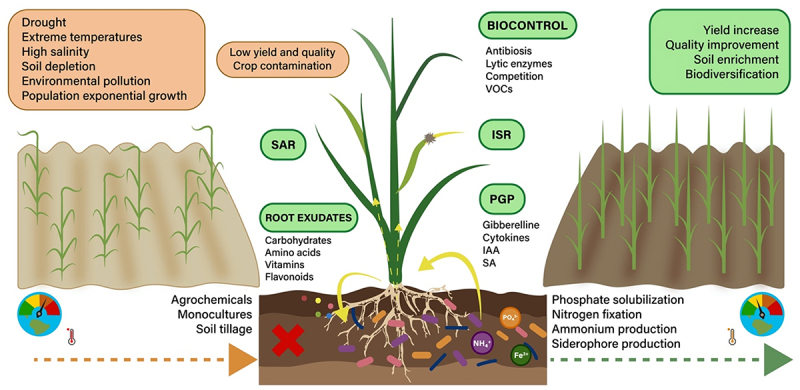

The world population has been rapidly increasing for many decades, and it is estimated to reach 9.7 billion by 2050, which is 34% higher than today, according to the United Nations.^1^ This accelerated growth has created issues in the global food supply and demand, forcing farmers to implement intensive agricultural practices that harm the environment, to increase crop yields, which are predicted to require an increment of at least 70%.^2,3^ These non-sustainable agricultural practices have led to diverse negative impacts, such as the detriment of agricultural soils, as well as their desertification and erosion; the emission of greenhouse gases; the pollution and eutrophication of water reservoirs; a critical decrease in native microbial diversity, and the excessive release of agrochemicals to the environment.^4,5^ Furthermore, the massive application of agrochemicals interferes with the ability of soil microbiome to conduct nutrient cycling.^6^ For this reason, for over three decades, the scientific community, governments, and general society have been focusing on developing sustainable agricultural practices to obtain highly productive crops while switching to more ecological alternatives to preserve the agroecosystem.^7,8^

During their life cycle, plants are subjected to a variety of biotic constraints caused by diverse pathogenic fungi, nematodes, viruses, and insects, as well as to several abiotic stresses, such as drought, high salinity, and extreme temperatures.^9^ Microbes associated with the plant can exert prejudicial, neutral, and beneficial relations with their host.^6^ A strategy that has already proven effective in increasing crop resilience to those risks, as well as promoting plant growth and yield, is the application of beneficial microorganisms to crops.^10,11^ This approach leads to numerous benefits, such as plant growth promotion by increasing nutrient bioavailability and water use efficiency, as well as by naturally suppressing pathogenic proliferation.^9,12^ Common biological processes mediated by microorganisms, which result in the promotion of plant growth, include nitrogen fixation, phosphate solubilization, plant growth regulators (phytohormones) production, and siderophore production, which increase nutrient availability for the plants. Bacteria with phosphate solubilization capabilities include the genus *Bacillus*, *Azotobacter*, *Microbacterium*, *Erwinia*, *Enterobacter*, *Flavobacterium*, *Rhizobium*, and *Pseudomonas*, among others,^13^ which have been reported to increase phosphorus availability by three or five times through a variety of mechanisms, such as solubilization, mineralization and mobilization, as well as by modifying root structure to increase the uptake of this nutrient.^14^ Nitrogen-fixing microbes include *Azotobacter*, *Azospirillum*, *Gluconacetobacter, Rhizobium*, and cyanobacteria.^13^ For non-leguminous crops (wheat, rice, maize), as they do not associate with rhizobia, the understanding of molecular mechanisms of root nodule symbioses allows the engineering of associative nitrogen fixation and/or free-living nitrogen fixation with beneficial bacteria, which contributes with up to 25% of the total amount of nitrogen in harvested grains.^15^ Furthermore, a native bacterial consortium exhibited the ability to increase grain yield while decreasing the amount of nitrogen fertilizer by half on field trials performed on wheat crops in Mexico.^16^ Additionally, the soil microbiome is capable of producing plant growth regulators such as auxins, gibberellic acid, salicylic acid, and cytokinins.^6^ As evidenced, employing either beneficial microorganisms or microbial-produced compounds (e.g. lipochitooligosaccharides, flavonoids, phenolics, alkaloids, and carotenoids) is an important strategy for increasing soil fertility as they improve the availability and uptake of essential nutrients such as nitrogen, phosphorus, zinc, iron and potassium and act as biostimulants, promoting plant development. Furthermore, several microbial compounds have been reported to enhance plant tolerance to abiotic stress, by scavenging reactive oxygen species, diminishing ethylene concentration on the stressed plant (through ACC deaminase), and/or producing exopolysaccharides and osmolytes, among other mechanisms.^17^ The application of these bioactive compounds on agricultural fields represents a promising research direction and requires extensive testing to ensure their effectiveness and safety. On the other hand, the inhibition of phytopathogens by plant microbiome is mediated mainly by the production of antibiotics, hydrogen cyanide, phenazines, lytic enzymes, siderophores, volatile compounds, and by competition for nutrients and space.^13,17^ Furthermore, the beneficial microbiome stimulates the plant defense system by a mechanism known as induced systemic resistance (ISR), which together with the plant systemic acquired resistance (SAR) increases plant resistance to biotic and abiotic stresses.^6,13^

The positive effects aforementioned result from the intimate association between plants and beneficial microorganisms, creating a symbiotic interaction with mutual benefits for both organisms, which plays a critical role in plant communities’ establishment, health, and nutrition.^13,18^ For instance, the root exudates provide essential nutrients and carbon sources for microbial communities to thrive,^19,20^ and reciprocally, the existing microorganisms facilitate nutrient uptake and stimulate host plant defenses against potential pathogens.^21^ However, only a few plant-microbial symbiotic interactions have been studied in-depth due to the limited number of cultivable microbes, which represent barely about 5%.^12^ For this reason, the application of omic sciences and genome mining in the study of microbiomes has allowed huge advances in the understanding of microbial communities, their composition, mechanisms of interaction, and putative bioactive compounds.^6^ Currently, most in-depth studies on plant-microbiome interactions, at a genomic, transcriptomic, or molecular level, are limited to model plants, such as *Arabidopsis*.^18^ However, the information obtained from these model systems has led the way and opened opportunities to apply this knowledge, as interest in elucidating the intricate mechanisms of these interactions with other plants of agricultural importance has recently grown among the scientific community.

As climate change progresses, the existing interaction mechanisms will evolve and complexify,^22,23^ and the current knowledge may lose accuracy. Hence, it is imperative to design the ongoing research taking into account not only the existing climatic conditions but also the predicted future scenarios which anticipate global temperature increases from 1.5 to 2°C above pre-industrial level, resulting in extreme climate events such as drought, heat waves, and floods.^24–26^ For this, modeling of microbiome behaviors using whole-population sequencing (metagenomics) and metatranscriptomic data is an innovative tool for predicting shifts in interaction mechanisms, as these techniques allow for the characterization of the whole diversity and its potential function in the microbiome, without the limitation of isolation and culturing.^27^ The knowledge gap in this field would require the application of multiple disciplines, including biochemical, microbiological, microscopic, genetic, and omic techniques, to fully understand these complex relationships and utilize them for sustainable agriculture and global food safety.^28^

Scientific collaborations of research teams around the world are essential to achieve the main goals of sustainable agriculture, which are increasing crop productivity, decreasing environmental impact, and preserving microbial diversity, in an ecologically friendly and economically sustainable way. Sharing knowledge and breakthroughs in the field will facilitate the development of effective microbial inoculants worldwide. Moreover, communication channels must be created with farmers, decision-makers, and general society to better inform them on the current agricultural problems we face and the innovative strategies that are being researched. This will also allow us to disregard the misinformation about the use of beneficial microorganisms for sustainable agriculture and create awareness about the loss of fertile, cultivable land for future generations. Additionally, the optimal strategy to better exploit beneficial microbial symbiosis to improve crop productivity has to be adapted to each specific crop’s needs and site of application. We consider that the use of cutting-edge technologies is crucial for ensuring a correct assessment of soil physico-chemical and microbial characteristics, to correctly meet its requirements when applying beneficial microorganisms and/or its derived compounds. By taking these approaches we may considerably decrease the current inconsistency between laboratory and field efficacy of microbial inoculants. Thus, this article compilation aims to gather state-of-the-art research and new insights into the plant-microbial symbiotic relationships, to harness their potential in sustainable agriculture. Such research includes but is not restricted to the use of novel techniques for the elucidation of these interactions, such as genomic, transcriptomic, and metabolomic analyses, molecular, biochemical, and metabolic mechanisms, effects of symbionts in plant resistance and yield, microbiome interactions, and networks, modeling, and prediction of such interactions for future climate conditions, bioprospection and bioformulation advances and technologies, among others. This information will allow to optimization of the application of symbiotic relationships between crops and their associated microbes and/or derived molecules to improve soil fertility and decrease the reliance on chemical fertilizers. As guest editors, we hope that the information compiled in it will inspire research collaborations and will contribute to a better understanding of the complex symbiotic interactions between plants and microbes, toward improving yield productivity, quality, and sustainability of crops.

## References

[1] UN Department of Economic and Social Affairs - Population Division. Global population growth and sustainable development. *United Nations*; 2021. www.unpopulation.org.

[2] de Los Santos-Villalobos S, Parra-Cota FI. Current trends in plant growth-promoting microorganisms research for sustainable food security. Curr Res Microbial Sci. 2021;2(October 2020):100016. doi:10.1016/j.crmicr.2020.100016. PMC8714767 35028625

[3] Tripathi AD, Mishra R, Maurya KK, Singh RB, Wilson DW. Estimates for world population and global food availability for global health. InThe role of functional food security in global health. Academic Press; 2018. pp. 3–4. doi:10.1016/B978-0-12-813148-0.00001-3.

[4] Ibarra-Villarreal AL, Parra-Cota FI, Yepez EA, Gutiérrez-Coronado MA, Valdez-Torres LC, de Los Santos-Villalobos S. Impact of a shift from conventional to organic wheat farming on soil cultivable fungal communities in the Yaqui Valley, Mexico. Impacto del cambio en el manejo del cultivo de trigo de convencional a orgánico sobre las comunidades fúngicas cultivables del. Agrociencia. 2020;54(5):643–659. https://dialnet.unirioja.es/servlet/articulo?codigo=7542484&info=resumen&idioma=ENG.

[5] Porter SS, Sachs JL. Agriculture and the disruption of plant–microbial symbiosis. Trends Ecol Evol. 2020;35(5):426–439. doi:10.1016/J.TREE.2020.01.006. 32294424

[6] Suman J, Rakshit A, Ogireddy SD, Singh S, Gupta C, Chandrakala J. Microbiome as a key player in sustainable agriculture and human health. Front Soil Sci. 2022;2:821589. doi:10.3389/fsoil.2022.821589.

[7] Aznar-Sánchez JA, Piquer-Rodríguez M, Velasco-Muñoz JF, Manzano-Agugliaro F. Worldwide research trends on sustainable land use in agriculture. Land Use Policy. 2019;87:104069. doi:10.1016/j.landusepol.2019.104069.

[8] Calicioglu O, Flammini A, Bracco S, Bellù L, Sims R. The future challenges of food and agriculture: an integrated analysis of trends and solutions. Sustainab (Switzerland). 2019;11(1):222. doi:10.3390/su11010222.

[9] Kumar A, Patel JS, Meena VS, Srivastava R. Recent advances of PGPR based approaches for stress tolerance in plants for sustainable agriculture. In: Biocatalysis and agricultural biotechnology. Vol. 20. Elsevier; 2019. p. 101271. doi:10.1016/j.bcab.2019.101271.

[10] Naik K, Mishra S, Srichandan H, Singh PK, Sarangi PK. Plant growth promoting microbes: potential link to sustainable agriculture and environment. Biocatal Agric Biotechnol. 2019;21:101326. doi:10.1016/J.BCAB.2019.101326.

[11] Poudel M, Mendes R, Costa LAS, Bueno CG, Meng Y, Folimonova SY, Garrett KA, Martins SJ. The role of plant-associated bacteria, fungi, and viruses in drought stress mitigation. In: Frontiers in microbiology. Vol. 12. Frontiers Media S.A; 2021. p. 743512. doi:10.3389/fmicb.2021.743512. 34759901 PMC8573356

[12] Lyu D, Msimbira LA, Nazari M, Antar M, Pagé A, Shah A, Monjezi N, Zajonc J, Tanney CAS, Backer R, et al. The coevolution of plants and microbes underpins sustainable agriculture. Microorgan. 2021;9(5):1036. doi:10.3390/MICROORGANISMS9051036. PMC8151373 34065848

[13] Malgioglio G, Rizzo GF, Nigro S, du Prey VL, Herforth-Rahmé J, Catara V, Branca F. Plant-microbe interaction in sustainable agriculture: the factors that may influence the efficacy of PGPM application. Sustainab. 2022;14(4):2253. doi:10.3390/SU14042253.

[14] Bargaz A, Elhaissoufi W, Khourchi S, Benmrid B, Borden KA, Rchiad Z. Benefits of phosphate solubilizing bacteria on belowground crop performance for improved crop acquisition of phosphorus. In: Microbiological research. Vol. 252. Urban & Fischer; 2021. p. 126842. doi:10.1016/j.micres.2021.126842. 34438221

[15] Pankievicz VCS, Irving TB, Maia LGS, Ané JM. Are we there yet? The long walk towards the development of efficient symbiotic associations between nitrogen-fixing bacteria and non-leguminous crops. BMC Biology BioMed Central. 2019;17(1):1–17. doi:10.1186/s12915-019-0710-0. PMC6889567 31796086

[16] Ibarra-Villarreal A, Villarreal-Delgado L, Parra-Cota MF, Yepez FI, Guzmán EA, Gutierrez-Coronado MA, Valdez LC, Saint-Pierre C, de Los Santos-Villalobos S. Effect of a native bacterial consortium on growth, yield, and grain quality of durum wheat (Triticum turgidum L. subsp. durum) under different nitrogen rates in the Yaqui valley, Mexico. Plant Signal Behav. 2023;18(1). doi:10.1080/15592324.2023.2219837. PMC10730153 37294039

[17] Naamala J, Smith DL. Relevance of plant growth promoting microorganisms and their derived compounds, in the face of climate change. Agronomy. 2020;10(8):1179. doi:10.3390/AGRONOMY10081179.

[18] Santoyo G. How plants recruit their microbiome? New insights into beneficial interactions. J Adv Res. 2022;40(December):45–58. doi:10.1016/j.jare.2021.11.020. 36100333 PMC9481936

[19] Sasse J, Martinoia E, Northen T. Feed your friends: do plant exudates shape the root microbiome? Trends Plant Sci. 2018;23(1):25–41. doi:10.1016/j.tplants.2017.09.003. 29050989

[20] Upadhyay SK, Srivastava AK, Rajput VD, Chauhan PK, Bhojiya AA, Jain D, Chaubey G, Dwivedi P, Sharma B, Minkina T. Root exudates: mechanistic insight of plant growth promoting rhizobacteria for sustainable crop production. In: Frontiers in microbiology. Vol. 13. Frontiers Media S.A; 2022. p. 916488. doi:10.3389/fmicb.2022.916488. 35910633 PMC9329127

[21] Santoyo G, Guzmán-Guzmán P, Parra-Cota FI, de Los Santos-Villalobos S, Del Orozco-Mosqueda MC, Glick BR. Plant growth stimulation by microbial consortia. Agronomy. 2021;11(2):219. doi:10.3390/agronomy11020219.

[22] Wahid F, Sharif M, Ali A, Fahad S, Adnan M, Noor M, Mian IA, Khan IA, Alam M, Saeed M, et al. Plant-microbes interactions and functions in changing climate. InEnvironment, climate, plant and vegetation growth. Springer International Publishing; 2020pp. 397–419. doi:10.1007/978-3-030-49732-3_16.

[23] Yuan MM, Guo X, Wu L, Zhang Y, Xiao N, Ning D, Shi Z, Zhou X, Wu L, Yang Y, et al. Climate warming enhances microbial network complexity and stability. Nat Clim Chang. 2021;11(4):343–348. doi:10.1038/s41558-021-00989-9.

[24] Cavicchioli R, Ripple WJ, Timmis KN, Azam F, Bakken LR, Baylis M, Behrenfeld MJ, Boetius A, Boyd PW, Classen AT, et al. Scientists’ warning to humanity: microorganisms and climate change. Nat Rev Microbiol. 2019;17(9):569–586. Nature Publishing Group. doi:10.1038/s41579-019-0222-5. 31213707 PMC7136171

[25] Cogato A, Meggio F, Migliorati MDA, Marinello F. Extreme weather events in agriculture: a systematic review. Sustainabil. 2019;11(9):2547. doi:10.3390/SU11092547.

[26] Li K, Pan J, Xiong W, Xie W, Ali T. The impact of 1.5 °C and 2.0 °C global warming on global maize production and trade. Sci Rep. 2022;12(1):1–14. doi:10.1038/s41598-022-22228-7. 36241905 PMC9568596

[27] Iquebal MA, Jagannadham J, Jaiswal S, Prabha R, Rai A, Kumar D. Potential use of microbial community genomes in various dimensions of agriculture productivity and its management: a review. In: Frontiers in microbiology. Vol. 13. Frontiers Media S.A; 2022. p. 708335. doi:10.3389/fmicb.2022.708335. 35655999 PMC9152772

[28] de Los Santos-Villalobos S, Díaz-Rodríguez AM, Ávila-Mascareño MF, Martínez-Vidales AD, Parra-Cota FI. COLMENA: a culture collection of native microorganisms for harnessing the agro-biotechnological potential in soils and contributing to food security. Diversity. 2021;13(8):337. Multidisciplinary Digital Publishing Institute. doi:10.3390/d13080337.

